# Determinants of Green Consumer Loyalty in Sustainable Fashion: The Mediating Role of Green Brand Trust

**DOI:** 10.3390/bs16071207

**Published:** 2026-07-17

**Authors:** Ganesh Dash

**Affiliations:** College of Administrative and Financial Sciences, Saudi Electronic University, Riyadh 11673, Saudi Arabia; g.dash@seu.edu.sa

**Keywords:** green consumer loyalty, green brand quality, green brand price, green brand image, green brand trust, S-O-R, theory of loyalty

## Abstract

Consumer loyalty has become crucial in the sustainable fashion market for continued growth and sustainable competitive advantage. Building upon the theory of loyalty and the Stimulus–Organism–Response (S-O-R) framework, this study investigates how green brand quality, green brand price, and green brand image influence green consumer loyalty. Additionally, this study evaluates the mediating role of green brand trust. Data were collected via an online survey of 340 sustainable fashion consumers. Structural equation modeling (SEM) was used to test direct and mediating hypotheses. The findings reveal that green brand quality and green brand image influenced green consumer loyalty. However, green product price had no effect. Additionally, green brand trust strongly influenced green consumer loyalty. It also played a mediating role between quality, image, and loyalty. Ultimately, this study positions brand image, quality, and trust as foundational mechanisms in sustainable consumer behavior, especially green consumer loyalty.

## 1. Introduction

Given surging carbon emissions and environmental degradation, it is crucial that we thoroughly understand the various elements of eco-friendly, sustainable consumer behavior. Extant literature underscores sustainable living as the most effective strategy for environmental preservation (Yadav et al., 2025; McNeill & Moore, 2015). As in other sectors, the fashion industry has also seen significant progress in sustainable fashion (Yadav et al., 2025; Kong et al., 2016). Following the COVID-19 pandemic, implementing sustainable consumption practices is more critical than ever (Dash et al., 2024). Sustainable fashion focuses on creating, producing, and wearing clothing in ways that benefit society and the industry while protecting the environment. Eco-friendly materials, ethical production, and the circular economy are essential to sustainable fashion (Mukendi et al., 2020). However, green consumption is not enough; repeated purchases are essential, and hence green consumer loyalty has become crucial (Uikey et al., 2026; Chen, 2010). Green loyalty fuels sustainable fashion growth. Authentic, transparent, and circular practices in sustainable fashion drive repeat business. Consumers buy again when they trust the sustainability claims, share brand values (of the seller), and obtain rewards for recycling the purchased products (Uikey et al., 2026; Yadav et al., 2025).

Consumer loyalty is fundamentally understood as a deep commitment to repurchasing a preferred product over time (Oliver, 1999, 1997). Historically, brand loyalty was divided into two dimensions: behavioral loyalty (the repeated purchase of a product) and attitudinal loyalty (the mental preference and emotional attachment to a brand). It was just a mix of what consumers did and how they felt. Green consumer loyalty refers to a customer’s dedicated commitment to consistently buy a specific eco-friendly product, alongside their intention to maintain a lasting relationship with an environmentally conscious brand (Gelderman et al., 2021). High product quality directly fosters customer retention and repeat purchases. Shoppers stay loyal when brands consistently deliver durable goods and reliable service. Consistent performance builds trust, high durability justifies the price, and dependable products eliminate customer risk (Chang & Fong, 2010; Kotler et al., 2005). It is more crucial in sustainable fashion. Similarly, green product price also enhances consumer loyalty, especially among Gen Z and millennials (Uikey et al., 2026; Yadav et al., 2025). Modern environmentally conscious consumers actively want to reduce their environmental impact and are willing to pay more for sustainable options (Gelderman et al., 2021; Juwaheer et al., 2012). A strong green brand image directly boosts customer retention and repeat sales. Consumers remain loyal when a company is perceived as prestigious, socially responsible, and a reflection of consumers’ personal identity (Rahman & Nguyen-Viet, 2023; Gelderman et al., 2021; Chang & Fong, 2010). Based on the discussion above, this study aims to achieve the following objectives:

*RO1: To examine the influence of three antecedents—green product quality (GPQ), green product price (GPP), and green brand image (GBI)—on green consumer loyalty (GCL).*
*RO2: To evaluate the mediating role of green brand trust (GBT) in these relationships mentioned above.*

The remainder of this paper is structured as follows: Section 2 and Section 3 elaborate the theoretical background and literature review; Section 4 outlines the research methodology; and Section 5 presents the data analysis and empirical results. Finally, the study concludes with a discussion and implications.

## 2. Theoretical Background

The study relies upon two major theories and frameworks in behavioral sciences. The first is the Four-Stage Theory of Loyalty (Oliver, 1997), and the guiding framework for the proposed model is the stimulus-organism-response (S-O-R) model (Mehrabian & Russell, 1974). Oliver (1997, 1999) outlined the step-by-step evolution of consumer loyalty. Rather than viewing consumer retention as a binary state, this framework demonstrates that loyalty matures gradually through a behavioral sequence of cognition, affect, conation, and action (Oliver, 1997, 1999). Based on this theory, green product quality, green product price, and green brand image are considered as antecedents of green consumer loyalty. Developed by Mehrabian and Russell (1974), the S-O-R framework models how individuals behave when exposed to varied environmental stimuli. The core premise is that external environmental cues shape a person’s cognitive and emotional states, which, in turn, drive their final behavioral responses (Dash et al., 2026; Rahman & Nguyen-Viet, 2023).

**Figure 1:**
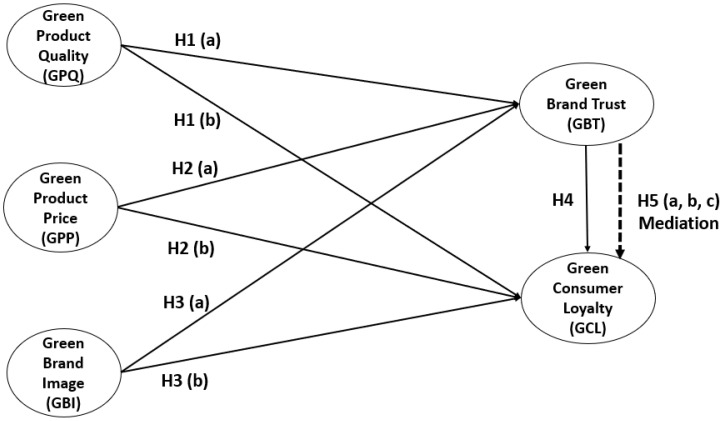
Conceptual model.

In the current work, green product quality (GPQ), green product price (GPP), and green brand image (GBI) serve as environmental stimuli (S). Meanwhile, green brand trust (GBT) represents the organism (O), and green consumer loyalty (GCL) acts as the final behavioral response (R), as illustrated in Figure 1.

## 3. Literature Review and Hypotheses Formulation

### 3.1. Green Product Quality, Green Brand Trust, and Green Consumer Loyalty

According to Abdul-Muhmin (2002), product quality is a multidimensional construct encompassing packaging, design, specific features, and warranties (Chang & Fong, 2010). Maximizing these quality dimensions helps firms achieve broader consumer adoption. It fosters stronger satisfaction among supply chain partners, including retailers and wholesalers (Chang & Fong, 2010).

Green product quality has a decisive role in building green brand trust (Chen & Chang, 2013). High product quality yields several business benefits, e.g., word-of-mouth (positive), reduced consumer management costs, increased sales volume, and higher price premiums (Chen & Chang, 2013; Sweeney et al., 1999; Qualls & Rosa, 1995). Therefore, in the modern eco-friendly marketplace, green product quality has become a critical determinant of brand success. Consumer trust heavily relies on the product quality (perceived) as a behavioral dimension (Chen & Chang, 2013; Kardes et al., 2004). McKnight et al. (1998, 2004) highlight that perceived quality not only builds consumer trust but also acts as one of the primary factors driving it. Based on the above academic inferences, it is proposed that:

**H1(a):**  *Green product quality is positively associated with green brand trust.*

Chang and Fong (2010) claim that product quality directly influences firm performance and is intrinsically tied to consumer satisfaction and loyalty (Eskildsen et al., 2004). This body of research indicates that sustaining high product quality consistently satisfies consumers, thereby fostering long-term consumer loyalty (Chang & Fong, 2010; Kotler et al., 2005). Prior research establishes that superior product quality drives consumer satisfaction and enhanced loyalty (Gelderman et al., 2021). When applied to sustainable (green) contexts, this quality dimension becomes green product quality, characterized by distinct environmental attributes and eco-friendly benefits. To build sustainable competitive advantage and foster consumer loyalty, companies must look beyond merely integrating environmental concepts into product design or packaging for differentiation. Instead, they must align their offerings with their consumers’ actual environmental needs and values (Chang & Fong, 2010; Shrivastava, 2018). Based on the above academic inferences, it is proposed that:

**H1(b):**  *Green product quality is positively associated with green consumer loyalty.*

### 3.2. Green Product Price, Green Brand Trust, and Green Consumer Loyalty

Konuk (2017) defines product price as the consumer’s cognitive evaluation and emotional response regarding whether the discrepancy between a seller’s price and a competitor’s price is reasonable, acceptable, or justifiable (Gelderman et al., 2021). Product pricing for green or sustainable products is called green product pricing.

Brand trust, especially for green products, is crucial for any business to maintain a healthy relationship with consumers. Chaudhuri and Holbrook (2001) highlight that an operational definition of trust fundamentally incorporates consumer perceptions of reliability, safety, and honesty. Consumers are environmentally aware and knowledgeable enough to verify a brand’s environmental credentials. Previous literature demonstrates a positive relationship between these two variables (Konuk, 2017; Weisstein et al., 2013). Pricing of the green product plays a huge role in enhancing brand trust. Accordingly, it is proposed that:

**H2(a):**  *Green product price is positively associated with green brand trust.*

Green products command higher prices due to significant costs incurred to enhance sustainability and environmentally friendly attributes (Suki, 2017). However, it also shows the product’s quality. A significant segment of consumers is willing to pay a premium for eco-friendly goods, provided that this financial sacrifice is offset by perceived ecological and functional values (Gelderman et al., 2021; Cǎtoiu et al., 2010). In fact, a premium price set for green products enhances consumer satisfaction and loyalty, as modern consumers are concerned about the environmental impacts of their consumption and are willing to pay more for green products (Gelderman et al., 2021; Juwaheer et al., 2012). Hence, the pricing of the green products may lead to enhanced consumer loyalty. Accordingly, it is proposed that:

**H2(b):**  *Green product price is positively associated with green consumer loyalty.*

### 3.3. Green Brand Image, Green Brand Trust, and Green Consumer Loyalty

Keller (1993) defines brand image as “perceptions about a brand as reflected by the brand associations held in consumer memory.” Green brand image refers to the collection of consumer perceptions regarding a brand’s dedication to and responsibility for environmental issues (Rahman & Nguyen-Viet, 2023; Chen, 2009).

By fulfilling consumers’ ecological expectations and mitigating associated environmental challenges, a strong green brand image fosters consumer trust by lowering perceived risk (Rahman & Nguyen-Viet, 2023; Khandelwal et al., 2019; Chen, 2009). Establishing a green brand image has become a strategic imperative for organizations, driven by rising consumer environmental consciousness and increasingly stringent global regulatory frameworks (Rahman & Nguyen-Viet, 2023; Gelderman et al., 2021). This positive relationship between eco-friendly brand image and trust is well-documented in prior literature. Accordingly, it is proposed that:

**H3(a):**  *Green brand image is positively associated with green brand trust.*

Green brand image has a positive association with consumer satisfaction and loyalty (Rahman & Nguyen-Viet, 2023; Gelderman et al., 2021; Chang & Fong, 2010). In environmentally sensitive sectors, green brand image plays a significant role in boosting consumer loyalty, especially among consumers who prioritize sustainability. Furthermore, empirical evidence demonstrates that an eco-friendly brand image does more than satisfy consumers’ environmental expectations and green requirements; it also drives sales growth and strengthens market competitiveness. Additionally, consumer loyalty is positively affected (Bathmanathan & Hironaka, 2016; Chang & Fong, 2010). Accordingly, it is proposed that:

**H3(b):**  *Green brand image is positively associated with green consumer loyalty.*

### 3.4. Green Brand Trust and Green Consumer Loyalty

As environmental regulations tighten and consumer demand for sustainability grows, establishing green trust is increasingly critical for corporate success (Uikey et al., 2026; Chen & Chang, 2013). To gain a competitive advantage, firms should implement green marketing initiatives; these strategies address consumer environmental concerns by boosting perceived quality and lowering perceived risk (Chen, 2009). Green consumer loyalty is defined as a consumer’s commitment to repeatedly purchase a preferred eco-friendly product and their desire to sustain a long-term relationship with an environmentally responsible institution (Gelderman et al., 2021). A consumer’s loyalty is strengthened once trust in the brand is established. Green brand trust acts as a crucial driver of green brand loyalty. By validating a firm’s environmental claims, trust effectively mitigates consumer-perceived risk, which in turn fosters a sustained commitment to repeat green purchases (Uikey et al., 2026; Gelderman et al., 2021; Chen & Chang, 2013; Chen, 2009). Accordingly, it is proposed that:

**H4:**  *Green brand trust is positively associated with green consumer loyalty.*

In addition, green brand trust is used as a mediator between the three antecedents and the dependent construct, green consumer loyalty. Although it serves as an antecedent of consumer loyalty, it also serves as a mediator. The mediating role of green brand trust has been well researched in prior literature (Rahman & Nguyen-Viet, 2023; Hanaysha, 2022; Gelderman et al., 2021; Chen & Chang, 2013; Chang & Fong, 2010).

Accordingly, it is proposed that:

**H5(a):**  *Green brand trust mediates the relationship between green product quality and green consumer loyalty.*

**H5(b):**  *Green brand trust mediates the relationship between green product price and green consumer loyalty.*

**H5(c):**  *Green brand trust mediates the relationship between green brand image and green consumer loyalty.*

## 4. Methodology

### 4.1. Measurement

As the literature review and conceptual model suggested, this study uses five constructs. Measurement scales were sourced from prior studies and slightly modified to fit the specific context of this research. The constructs and the items (questionnaire), along with their sources, are provided in Table 1.

### 4.2. Sample and Data

Data collection took place from December 2025 to February 2026 across four major cities and their peripheries in India. A structured questionnaire with a five-point Likert scale, with 1 being “strongly disagree” and 5 being “strongly agree”. The questionnaire was distributed online. The chosen industry was sustainable fashion, and the target audience was fashion-savvy millennials; accordingly, some basic criteria were set: being a millennial born between 1981 and 1996 (aged 31–45), having purchased a sustainable fashion product (garments, shoes, accessories, and others) within the past three months, and demonstrating environmental consciousness. The age range of 31–45 years was set to ensure equitable distribution of age sub-groups. These strict filters targeted only environmentally conscious millennial frequent buyers of sustainable fashion products. The sampling frame was contacted via social media and email. According to the Raosoft (2025) sample size calculator, a sample size of 270–385 participants was required (at 90% to 95% confidence levels). Out of 415 questionnaires retrieved from the respondents, 340 passed preliminary data-cleaning checks and were retained for final analysis. A structured questionnaire with a five-point Likert scale, with 1 being “strongly disagree” and 5 being “strongly agree”. The questionnaire was distributed online. The demographic details of the sample are provided in Table 2.

## 5. Analysis and Results

### 5.1. Measurement Model

Following established structural equation modeling protocols (Ringle et al., 2024; Dash & Paul, 2021), the author evaluated the measurement model to confirm a distinct factor structure. A basic exploratory factor analysis (EFA) was conducted first using IBM SPSS 26 (IBM Corp., 2020). It provided a five-factor solution (80% of the variance explained), validating our proposed model. The details of the EFA are provided in Table 3.

Although PLS (variance) is used for SEM, it is not an ideal tool for the measurement model (covariance-based). For the measurement model evaluation, IBM SPSS Amos (Dash et al., 2026; Arbuckle, 2019) was used; for the final structural model, PLS was used. The confirmatory focus of the covariance tool (Amos), its detailed output of model fit indices, and the stringent conditions it imposes encourage authors to use it for confirmatory factor analysis. PLS (variance-based) tools (Smart PLS) are more focused on explained variance, suitable for the analysis of complex models (mediation-moderation), and slightly less stringent than CB-SEM. The measurement model evaluation indicated acceptable fit indices (CMIN/DF: 2.86, Goodness-of-fit index (GFI): 0.90, Adjusted goodness-of-fit Index (AGFI): 0.87, Standardized Root mean square residual (SRMR): 0.05, Root mean square error of approximation (RMSEA): 0.07, Tucker–Lewis index (TLI): 0.91, Normed fit index (NFI): 0.93, Comparative fit index (CFI): 0.93). Although gpp3 showed some issues, it was kept in the final analysis because the overall model was not significantly affected, and its factor loading was high in both EFA and CFA. Again, gbt4 had a low loading (0.575) in EFA, but it had a high loading in CFA; hence, it was kept. All five constructs demonstrated high internal consistency, with Cronbach’s alphas comfortably exceeding the 0.80 benchmark. Convergent validity was successfully established, as Composite Reliability (CR) values surpassed 0.80 and Average Variance Extracted (AVE) values exceeded 0.50 (Dash & Paul, 2021; Henseler et al., 2015; Hu & Bentler, 1999), as detailed in Table 4. To assess construct distinctiveness, a heterotrait-monotrait (HTMT) ratio analysis was performed (Henseler et al., 2015). The maximum observed HTMT ratio was 0.71 (Table 5), which remained well below the stringent 0.85 threshold. These combined assessments confirm that the measurement model is both reliable and valid, justifying subsequent structural hypothesis testing.

### 5.2. Common Method Bias (CMB)

CMB can be addressed in two ways: procedural and statistical (Podsakoff et al., 2003). Procedure-wise, anonymity was protected, and explanations of the statements with “no right or wrong answers” information were provided. This ensured unbiased responses. Statistics-wise, Harman’s single-factor test was applied, and no single latent variable had more than 50% of the total variance. Additionally, a marker-variable method was also adopted (Williams et al., 2010). No statistically significant relationship was observed between the outcome variables and the marker variable. Based on these insights, CMB was negated.

### 5.3. Structural Model

**Figure 2:**
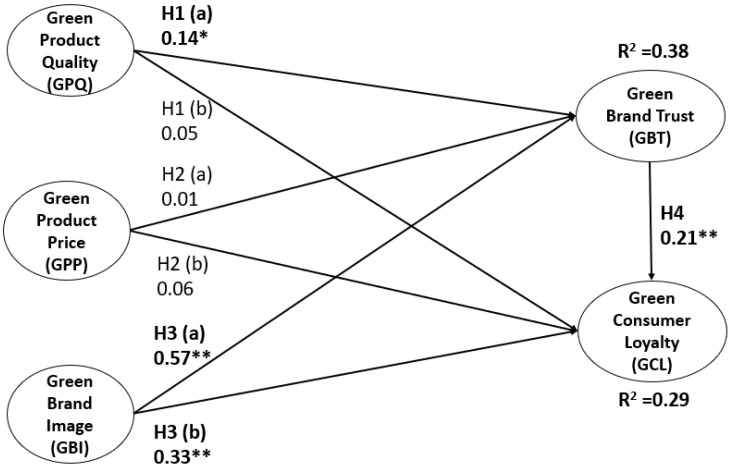
Direct Hypotheses. ** *p* < 0.01; * *p* < 0.05. Source: Smart PLS 4 outputs.

To evaluate the seven direct (H1–H4) and three mediation hypotheses, the structural model was analyzed using SmartPLS 4 (Ringle et al., 2024) with five constructs and 19 items. The model exhibited satisfactory explanatory power, with R^2^ values of 38% for Green Brand Trust (GBT) and 29% for Green Consumer Loyalty (GCL). As detailed in Figure 2, Green Product Quality (GPQ) and Green Brand Image (GBI) significantly and positively predicted GBT, supporting H1(a) (β = 0.14, *p* < 0.05) and H3(a) (β = 0.57, *p* < 0.01). GBI also demonstrated a positive relationship with GCL, confirming H3(b) (β = 0.33, *p* < 0.01). Furthermore, GBT was strongly associated with GCL, supporting H4 (β = 0.21, *p* < 0.01). Conversely, Green Product Price (GPP) did not show a significant relationship with either GBT or GCL, resulting in the rejection of those hypotheses.

### 5.4. Green Brand Trust as a Mediator

A mediation analysis was performed using SmartPLS 4.0, with the results summarized in Table 6. A bootstrapping procedure with 5000 resamples was employed to estimate 95% bias-corrected confidence intervals.

The analysis revealed that Green Brand Trust (GBT) fully mediated the relationship between Green Product Quality (GPQ) and Green Consumer Loyalty (GCL), as only the indirect effect reached statistical significance; thus, (H5(a)) was supported. Conversely, GBT did not mediate the relationship between GPP and GCL (H5(b)), as both the direct and indirect effects were statistically nonsignificant. Finally, GBT partially mediated the relationship between GBI and GCL, with both the direct and indirect effects significant, thereby supporting H5(c).

## 6. Discussion and Implications

This empirical study investigates how the three antecedents—GPQ, GPP, and GBI—influence GCL in the sustainable fashion industry. Grounded in Oliver’s (1997) four-stage theory of loyalty, this research uses the stimulus–organism–response (S-O-R) model (Mehrabian & Russell, 1974) to assess the proposed relationships, ultimately building on and extending previous quantitative insights into sustainable fashion. The role of GBT as a mediator is also evaluated.

First, the analysis revealed that GPQ has a significant positive association with GBT, but not with GCL. This means that green product quality enhances green trust. The findings are in line with previous literature. Chen and Chang (2013) found the same result in their study on Taiwanese consumers with prior experience purchasing consumer electronics and IT products. Higher product quality not only boosts customer satisfaction but also strengthens customer trust in the brand. Conversely, poor product quality damages the trust in the brand (Yeh & Li, 2009; Pavlou & Gefen, 2004; Garbarino & Johnson, 1999). However, GPQ did not have a significant impact on GCL. This finding contrasts with previous findings. It was widely accepted that green product quality has a direct impact on customer satisfaction and green loyalty (Gelderman et al., 2021; Chen & Chang, 2013; Chang & Fong, 2010). The current findings might be due to multiple reasons that can be explored in future research, e.g., a lack of proper understanding of the construct by the chosen sample; the socio-economic characteristics of the sample; the understanding of loyalty through satisfaction, and so on.

Second, in the current study, GPP (green product price) does not affect either GBT or GCL. The extant literature found two contrasting results on the ‘willingness to pay’ element for the consumers. Many studies reported that consumers did not bother to pay more for green products (Kordshouli et al., 2015; Chen, 2010), whereas others found that they did (Cheema et al., 2015). Gelderman et al. (2021) highlighted the importance of ‘price fairness’ in this context. Consumers are ready to pay more provided the perceived value of the product matches the environmental value additions. In this study, the influence was negligible. There are multiple reasons for this, and it can be the subject of future research. Some people suggest that price might no longer be the most important factor in green marketing, but that might not be entirely true. It is probably more accurate to say that while price still matters a lot, it is no longer the only thing consumers care about, and other factors also play important roles. The current sample consists of highly educated millennials who are well aware of the concept of sustainability. As environmentally conscious consumers, they know the real value of sustainable fashion and its positive contributions to the environment. Hence, by default, they expect a higher price for sustainable fashion products, and it does not influence their trust or loyalty.

Third, green brand image (GBI) was the most impactful antecedent of both GBT and GCL. While a general brand image relies on the collective consumer perceptions and mental associations tied to a company (Keller, 1993), a green brand image narrows that focus to how consumers evaluate the brand’s environmental stewardship and ecological commitment (Rahman & Nguyen-Viet, 2023; Chen, 2009). GBI has a strong association with GBT. This finding aligns with almost all previous literature (Rahman & Nguyen-Viet, 2023; Gelderman et al., 2021; Khandelwal et al., 2019; Chen, 2009). The perception of a brand’s pro-environmental image strongly influences eco-sensitive consumers. Nowadays, consumers are well aware of global environmental norms and guidelines, and, as a result, GBI is crucial for gaining consumer trust (Rahman & Nguyen-Viet, 2023). With a higher price and greater risks involved, GBI acts as insurance for green consumers. Similarly, GBI has a significant positive association with GCL. This finding also aligns with most previous studies’ findings (Rahman & Nguyen-Viet, 2023; Gelderman et al., 2021; Chang & Fong, 2010). GBI boosts customer satisfaction, enhances GCL, and reduces risk perception while providing a sustainable competitive advantage (Gelderman et al., 2021; Chang & Fong, 2010; Abdullah et al., 2000). GCL is a byproduct of green customer satisfaction, and both are considered simultaneously. Overall, the green brand image is most crucial in shaping the trust and loyalty of modern, environmentally conscious consumers.

Finally, the influence of GBT on GCL was assessed. It was positive and significant. Its role as a mediator was also assessed. The finding aligns with extant literature (Uikey et al., 2026; Gelderman et al., 2021; Chen & Chang, 2013; Chen, 2009). Transparent and genuine communication about sustainability strengthens green consumer loyalty. At the same time, trust in a company’s environmental claims remains a powerful indicator of the same. Proper brand communication reflects trust and influences GCL (Uikey et al., 2026; Lim & Jiang, 2024). It is particularly vital in high-stakes, high-involvement environments (risky) where consumers encounter heightened financial and functional risks (Uikey et al., 2026; Rahman & Nguyen-Viet, 2023). Under these conditions, establishing trust is essential to counter consumer skepticism and alleviate anxieties about greenwashing. The role of GBT as a mediator is equally crucial (Uikey et al., 2026). In this study, GBT appeared to mediate between GPQ, GBI, and GCL, whereas no mediation was found for GPP. The negligible influence of GPP on both GBT and GCL is already explained above. However, GBT has a strong role as a mediator for the other two. The brand’s real value perception among green consumers is ensured by strong GBT (Nguyen-Viet et al., 2024). It is vital to explore how green brand trust converts green perceived value (represented through GBI) into both word-of-mouth promotion and enduring green customer loyalty (Uikey et al., 2026). Both GPQ and GBI are well-supported by GBT in ensuring GCL in the long term.

### 6.1. Theoretical Implications

The current study has numerous theoretical contributions to the field of consumer behavior. First, PLS-SEM is highly suited for prediction and composite modeling. In contrast, CB-SEM is the standard for theoretical confirmation and model fit (Dash & Paul, 2021). Both methods were combined, which strengthens the empirical findings. It enhances the reproducibility of investigations into sustainability and consumer behavior models. Second, the use of the theory of loyalty and the S-O-R model in the context of sustainable fashion opens a new frontier that can scale up current sustainable fashion studies. The application of the S-O-R model in consumer behavior with a sustainability context adds value to the framework. Third, although the early stages of the four-stage loyalty theory are explored as antecedents, this opens the door to the advanced stages of loyalty and their application in sustainable consumer behavior, especially green consumer loyalty. Fourth, the use of green brand trust as both a mediator and an antecedent of green consumer loyalty adds immense value to trust theories—by generating new interest and offering new dimensions. Finally, the limited role of green product price prompts a closer examination of how pricing influences eco-friendly consumer choices. It does not align well with many prior theories, and it needs further research, as modern, eco-sensitive consumers might not see price as a barrier. Rather, price fairness has become an important indicator. Theories on price fairness in sustainable consumer behavior have to be advanced.

### 6.2. Practical Implications

Furthermore, this study’s findings have several practical implications. First, it provides a clear roadmap for sustainability-based marketers and policymakers, especially in the sustainable fashion industry. Industry leaders can understand the role of product quality and image in influencing loyalty among their eco-sensitive millennial consumers. Accordingly, product redesign or upgrade can be carried out. Second, they must note the limited role of pricing in the sustainable fashion sector and instead focus on other dimensions. However, price fairness must be visible to consumers, allowing them to compare the value added to environmental protection with the extra price they are paying. Third, it is quite clear that in sustainable marketing, consumers place great weight on green brand trust. Marketers and managers should identify the key components of brand trust and improve them to sustain consumer trust. Reliable, dependable branding is essential to the sustainable fashion industry. Trust shapes behavioral outcomes, and hence, the brand’s advertising or communication should be revisited to build long-term green consumer loyalty. Finally, most millennial consumers have a personal value (identity) system. Marketers must assess this and align the brand values or identity with those of their consumers.

## 7. Limitations and Future Research

Although this study addresses our main research questions, it has several limitations. First, the data were collected in a single phase, which may not reflect how people’s opinions change over time. Future studies should track participants over a longer period with longitudinal studies. Second, because participants completed the surveys themselves, there is a risk of biased responses. Although statistical controls were used to address this, future researchers should consider using interviews or a mix of surveys and interviews to achieve better results. A mixed-method study might provide the best solution. Third, this study was conducted only in India, so that the results may differ in other countries. Future research could compare two or more countries to see if the findings hold true globally. Fourth, the target sample consisted solely of millennials. Studies can add more age groups (generations) to check the multi-group findings. Finally, it was found that the price of green products did not affect consumers’ decisions. This was a surprising result, and future studies should look deeper into why shoppers feel this way about prices.

## Figures and Tables

**Table 1:** Constructs, Items, and Major Sources.

Construct	Items (Sustainable Fashion Products)	Source(s)
Green Product Quality (GPQ)	This (sustainable fashion) product (or/brand) meets or exceeds the requirements of environmental regulations. This (sustainable fashion) product (or/brand) uses the least resources and energy. This (sustainable fashion) product (or/brand) is easy to recycle, disassemble, and decompose, and can be reused.	(Gelderman et al., 2021; Suki, 2017; Chang & Fong, 2010)
Green Product Price (GPP)	The price of this green product is acceptable. The price of this green product is fair. The price of this green product is reasonable. The price of this green product is a premium, but it is within my budget.	(Gelderman et al., 2021; Konuk, 2017)
Green Brand Image (GBI)	The company’s green products are credible and stable. This company has the ability to meet consumers’ green needs. This company has a fine environmental reputation. This company has excellent performance in environmental management and green innovation.	(Gelderman et al., 2021; Suki, 2017; Chang & Fong, 2010)
Green Brand Trust (GBT)	I feel that this brand’s environmental commitments are generally reliable. I feel that this brand’s environmental performance is generally dependable. I feel that this brand’s environmental argument is generally trustworthy. This brand keeps promises and commitments for environmental protection.	(Rahman & Nguyen-Viet, 2023; Konuk et al., 2015)
Green Consumer Loyalty (GCL)	I will continue to shop with this company. I am willing to recommend my family, friends, and business relations to shop with this company. I can accept the higher price for green products, even though other (non-green) products are cheaper. I would be very disappointed if this brand were not available.	(Gelderman et al., 2021; Suki, 2017; Chang & Fong, 2010; Reid & Reid, 1994)

**Table 2:** Sample characteristics.

			Gender		Total
			Males	Females	
Age (years)	31–35	Count	61	71	132
		% of Total	17.9%	20.9%	38.8%
	36–40	Count	63	52	115
		% of Total	18.5%	15.3%	33.8%
	41–45	Count	59	34	93
		% of Total	17.4%	10.0%	27.4%
Total		Count	183	157	340
		% of Total	53.8%	46.2%	100.0%

Source: SPSS 26 outputs.

**Table 3:** EFA results.

Kaiser-Meyer-Olkin Measure of Sampling Adequacy—0.831 Bartlett’s Test of Sphericity—Approx. Chi-Square—3653.672 df 171 Sig. 0.000					
	Five-factor solution				
	1	2	3	4	5
gpq1					0.753
gpq2					0.857
gpq3					0.849
gpp1			0.701		
gpp2			0.793		
gpp3			0.903		
gpp4			0.780		
gbi1	0.828				
gbi2	0.790				
gbi3	0.707				
gbi4	0.750				
gbt1				0.678	
gbt2				0.794	
gbt3				0.847	
gbt4				0.575	
gcl1		0.776			
gcl2		0.875			
gcl3		0.791			
gcl4		0.764			

Source: SPSS outputs.

**Table 4:** Validity Analysis.

	CR	AVE	MSV	GPQ	GPP	GBI	GBT	GCL
GPQ	0.866	0.685	0.323	0.828				
GPP	0.860	0.607	0.319	0.568 ***	0.779			
GBI	0.852	0.590	0.488	0.244 ***	0.288 ***	0.768		
GBT	0.823	0.538	0.497	0.328 ***	0.244 ***	0.712 ***	0.734	
GCL	0.867	0.620	0.300	0.254 ***	0.216 ***	0.548 ***	0.505 ***	0.788

*** *p* < 0.001. Source: SPSS Amos outputs.

**Table 5:** HTMT Analysis.

	GPQ	GPP	GBI	GBT	GCL
GPQ					
GPP	0.649				
GBI	0.256	0.291			
GBT	0.316	0.265	0.713		
GCL	0.250	0.245	0.570	0.523	

Source: SPSS Amos outputs.

**Table 6:** Mediation Effects.

Relationship	Direct Effect	Indirect Effect	95% Confidence Interval of the Indirect Effect	Result
H5(a): GPQ→GBT→GCL	0.05	0.05 *	(0.012, 0.078)	Yes, Full
H5(b): GPP→GBT→GCL	0.06	0.01	(−0.020, 0.032)	No
H5(c): GBI→GBT→GCL	0.33 **	0.12 *	(0.036, 0.208)	Yes, Partial

** *p* < 0.01; * *p* < 0.05. Source: Smart PLS 4 outputs.

## Data Availability

The data will be made available on reasonable request.
